# Supplementary material for: Modulation of Reoviral Cytolysis (II): Cellular Stemness
Source: Viruses. 2023 Jun 29;15(7):1473. doi: 10.3390/v15071473 (PMC10386201; doi:10.3390/v15071473)
Supplement: Supplementary file 1 [file viruses-15-01473-s001.zip › viruses-2461661-supplementary.pdf]

Supplementary Materials:

The following are available online at www.mdpi.com/xxx/s1,

File S1

Primary Antibodies

Viral Antibodies
Polyclonal rabbit anti-reovirus (serotype 3 Dearing) antibody serum (Western blot 1:1000, immunofluorescence imaging 1:5000).

Loading controls
Mouse monoclonal Anti-β-Actin (Sigma #A2228, 1 μg/ml)
Mouse anti-actin antibody [ACTN05 (C4)] (Abcam #ab3280, 1:2000)

Yamanaka Factors
Anti-c-Myc Antibody (Novus Biologicals #NB600336 1:1000)
Anti-Sox2 Antibody (GeneTex #GTX101507, 1;5000)
Anti-KLF4 Antibody (Cell Signalling Technology #4038T, 1:1000)
Anti-Oct3/4 Antibody (Santa Cruz #sc5279, 1:100)

Liver organoid differentiation markers
Anti-HNF-4-alpha antibody (Abcam #Ab92378, 1:200)
Anti-SOX9 antibody (Abcam #Ab185966, 1:500)
Anti-Cytokeratin 7 antibody (Abcam #Ab68459, 1:200)
CYP3A4 Monoclonal Antibody (Thermo Fisher Scientific #MA5-17064, 1:200)
Anti-Collagen IV antibody (Abcam # ab6586)
Vimentin Monoclonal Antibody (Thermo Fisher Scientific # MA5-14564)
Anti-alpha smooth muscle Actin antibody (Abcam #ab7817)

Secondary antibodies

Western Blot
Goat anti-mouse IgG-HRP: (Santa Cruz #sc-2005, 1:7500)
Amersham ECL Mouse IgG, HRP-linked whole Ab from sheep (GE Healthcare Life Sciences #NA931, 1:7500)
Anti-rabbit IgG, HRP-linked Antibody (Cell signalling #7074, 1:1000)

Immunostaining
Donkey anti-rabbit-Alex647, (Thermo Fisher Scientific #A-31573, 1:5000)
Donkey anti-Mouse IgG (H+L) Highly Cross-Adsorbed Secondary Antibody, Alexa Fluor 546 (Thermo Fisher Scientific, #A10036, 1:1000)
Donkey anti-Rabbit IgG (H+L) Highly Cross-Adsorbed Secondary Antibody, Alexa Fluor 488 (Thermo Fisher Scientific, #A21206, 1:1000)

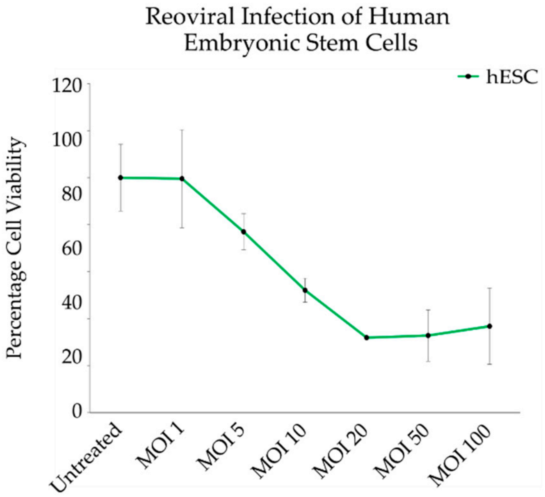

Figure S1: Reovirus mediated cytolytic killing in human embryonic stem cells.

Human embryonic stem cell lines H1 were infected with wild type reovirus (T3D). Crystal violet colorimetric analysis was used to determine cell viability three days post infection. Absorbance was read at 570 nm and normalised to untreated controls. The assay was performed in triplicate (n=3). Error bars represent standard deviation. Two-tailed paired student's t-test was used to compare treated groups to untreated controls.

A.

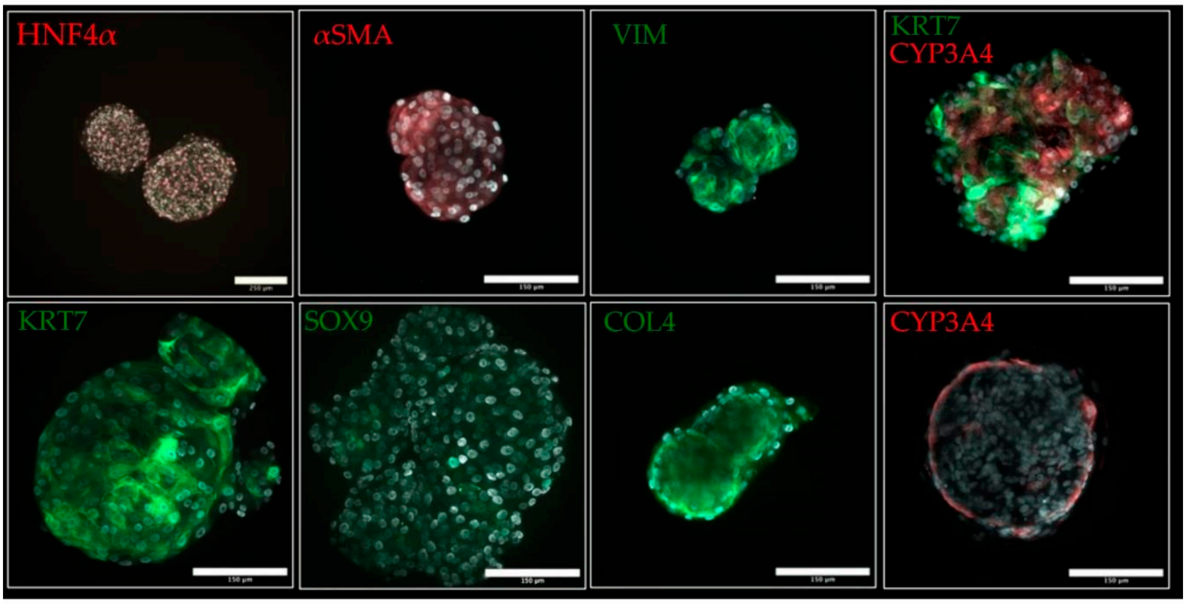

Figure S2: Confirmation of differentiated Human liver organoids

(A.) Differentiation of human embryonic and induced pluripotent stem cells was confirmed through immunofluorescence analysis. Expression of liver specific markers KRT7, CYP3A4, HNF4-α, VIM, COL4, SMAα and Sox9 was used to confirm the successful differentiation of liver organoids.

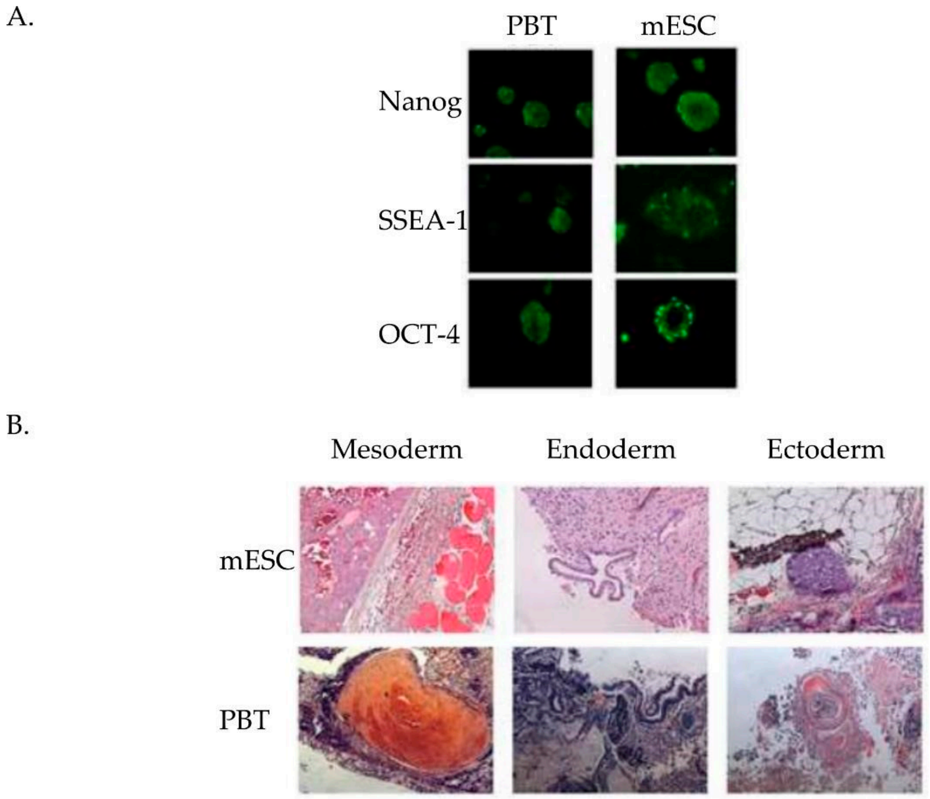

Figure S3: Characterisation of ipCSC.

(A.) Reprogramming of the ipCSCs from PiggyBac transposase vectors (PBT) was confirmed through the expression of pluripotency markers Oct, Nanog and SSEA-1. Expression was compared to mESC. (B.) Pluripotency was characterised in ipCSCs using the teratoma assay demonstrating cells are capable of differentiation into the three germ layers. Histochemical analysis demonstrated that ipCSCs could generate each of these cell types after teratoma formation.
